# Leptospiral Culture without 5’-Fluorouracil Revealed Improved *Leptospira* Isolation from Febrile Patients in North-Eastern Malaysia

**DOI:** 10.3390/ijerph17041307

**Published:** 2020-02-18

**Authors:** Amira Wahida Mohamad Safiee, Mohammad Ridhuan Mohd Ali, Mohd Hashairi Fauzi, Alwi Muhd Besari, Chan Yean Yean, Vasantha Kumari Neela, Nabilah Ismail

**Affiliations:** 1 Department of Medical Microbiology & Parasitology, School of Medical Sciences, Universiti Sains Malaysia, Health Campus, Kubang Kerian 16150, Kelantan, Malaysia; amirawahida1712@gmail.com (A.W.M.S.); yychan@usm.my (C.Y.Y.); 2 Bacteriology Unit, Infectious Disease Research Center (IDRC), Institute for Medical Research, Complex National Institutes of Health, Section U13 Setia Alam, Shah Alam 43200, Selangor, Malaysia; ridhuan.ali@gmail.com; 3 Department of Emergency Medicine, School of Medical Sciences, Universiti Sains Malaysia, Health Campus, Kubang Kerian 16150, Kelantan, Malaysia; hashairi@usm.my; 4 Department of Medicine, School of Medical Sciences, Universiti Sains Malaysia, Health Campus, Kubang Kerian 16150, Kelantan, Malaysia; dralwi@usm.my; 5 Department of Medical Microbiology & Parasitology, Faculty of Medicine and Health Sciences, Universiti Putra Malaysia, Serdang 43400, Malaysia; vasantha@upm.edu.my

**Keywords:** isolation, *Leptospira*, febrile illness, Malaysia

## Abstract

*Objectives:* Isolation of *Leptospira* by culture represents a definitive growth and confirmation of the disease, yet it is hampered with its nature of slow growth. With slight modification of culture method, the study aims to isolate and characterize *Leptospira* spp. from patients with acute febrile illness. *Methods:* A total of 109 blood samples were collected from patients with acute febrile illness that presented at the Emergency Department of Hospital Universiti Sains Malaysia, Malaysia. Clinical samples were subjected to *Leptospira* IgM Rapid test, microscopic agglutination test (MAT), isolation by culture method, and direct real-time PCR test. For leptospiral isolation, the samples (whole blood and deposit from spun plasma) were cultured into modified Ellinghausen McCullough Johnson Harris (EMJH) media with and without 5’-fluorouracil (5-FU). In every culture positive sample, partial 16S rRNA gene sequencing was performed for molecular identification of the isolates. Phylogenetic analysis was carried out to determine the genetic relatedness among the isolates. An inhibition of 5-FU study was performed on *Leptospira interrogans* serovar Canicola with different concentrations to compare the growth detection of the tested *Leptospira* with or without 5-FU within 7 days of incubation. *Results:* Leptospirosis was diagnosed in 14.7% of patients with acute febrile illness. Two *Leptospira* spp. (*n* = 2/109, 1.85%) were successfully isolated from whole blood and deposit from spun plasma samples. B004 and B208 samples were positive at day 11 and day 7, respectively, in EMJH media without addition of 5-FU. Sample B004 was identified as *Leptospira interrogans* and B208 as *Leptospira weilli*. Phylogenetic analysis confirmed that both of them were within pathogenic group and they were not related. The 5-FU inhibition study revealed that additional of 5-FU at final concentration of 200 µg/mL to EMJH media demonstrated an inhibitory effect on the growth of the tested strain *Conclusion:* Isolation of *Leptospira* spp. using EMJH media without addition of 5’-fluorouracil resulted in a better outcome. Two pathogenic *Leptospira* isolates were successfully cultivated from patients with acute febrile illness that were genetically not related.

## 1. Introduction

Leptospirosis is a global re-emerging zoonotic disease and occurs in Malaysia as an endemic disease [1]. It is caused by pathogenic *Leptospira* that can be isolated from both patients and the environment [2,3]. Leptospirosis cases rise during the floods and seasonal rainfall due to better survival of leptospires and wider transmission in the environment [4]. 

*Leptospira* gain entry into the human blood stream through skin abrasions, mucous membrane or cuts, directly from the carrier animals, or indirectly via a contaminated environment. Humans pose higher risk of infection while performing outdoor recreations, domestic activities, or occupations that require interaction with animals, freshwater, and soil [5]. The disease manifests from mild, influenza-like illness to severe complications which may involve renal and hepatic impairment, pulmonary distress, and death. During the acute phase of infection, patients may experience headache, fever, malaise, myalgia, conjunctival suffusion, and transient rash.

Acute febrile illness is one of the common symptoms for patients to seek treatment at the emergency department or hospital care [6,7]. In tropical or developing countries, symptoms of acute febrile illness are undifferentiated in many diseases such as hepatitis, meningitis, dengue, malaria, leptospirosis, typhoid, influenza, viral hemorrhagic fever, icteric fever, and rickettsiosis [6,7,8,9]. The undifferentiated acute febrile illness due to leptospirosis ranged from 1.1% to 29.5% [6,7,8,9,10,11,12]. 

Isolation of *Leptospira* by culture represents a definitive confirmation of the disease, yet it is hampered with its nature of slow growth that results in longer culture time [5]. In addition, positive culture rate for *Leptospira* is generally low [2,12,13]. However, once the isolate is obtained, it is very useful for molecular genotyping and epidemiology study. With slight modification of culture method and type of samples used, this study aimed to isolate *Leptospira* spp. from patients with acute febrile illness in a tertiary teaching hospital in north-eastern Malaysia.

## 2. Materials and Methods

### 2.1. Clinical Samples

Blood samples were obtained from patients presented at the Department of Emergency Medicine, Hospital Universiti Sains Malaysia (HUSM) from January 2017 until December 2017 with acute febrile illness, that fulfilled the inclusion and exclusion criteria. Acute febrile illness was defined as having fever at 38°C or higher, and not more than 14 days of illness [6]. Adult patients aged 18 years old and above that presented with fever for up to 14 days with temperature of 38 °C or more were included in the study. Those with confirmed cases or obvious sources of infection such as abscesses or other infectious diseases such as typhoid fever, dengue, or others, those with immunocompromised conditions such as cancer, HIV disease, or renal failure, or those already on antibiotics were excluded from the study. Written informed consent was obtained prior to sample collection. The blood samples for each patient comprised of 2 mL of blood in a plain tube for rapid *Leptospira* serology test, 2 mL of blood in an EDTA tube for molecular detection, and 4 mL of blood in a heparinized tube for isolation of *Leptospira.*

### 2.2. Rapid Leptospira Serology Test 

This *Leptospira* IgM Duo Rapid (ImmuneMed, Gangwon-do, Korea) is a semi-quantitative immunochromatography test for the detection of antibodies against *Leptospira* in the patients’ serum following manufacturer’s instruction. About 3 μL of serum sample was directly placed on the cassette hole followed by adding seven drops of sample diluents. Results were read and interpreted after 15 min. 

**Figure 1:**
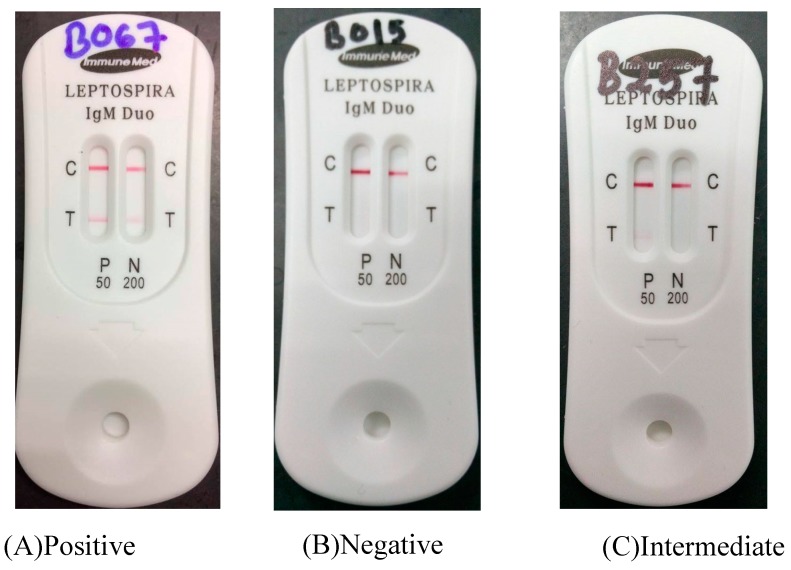
Example of the results for *Leptospira* IgM Duo Rapid test.

The test was valid if the red line appeared on the control line. Positivity was denoted by the presence of the red color line in T in both windows which are P 50 and N 200 (Figure 1). Then, the intermediate result was interpreted by the presence of the red color line in T in the P 50 window. Intermediate results were interpreted according to the region itself as Malaysia is an endemic region for leptospirosis. It was recommended by the manufacturer that the test result should be interpreted as intermediate or inconclusive rather than positive, and it was necessary to repeat the test within 2–7 days, if in an epidemic region, that results were interpreted as positive. IgM cut-off titer 50 and 200 were provided and could be used for probable diagnosis at any area in the world including epidemic and endemic regions. Figure 1 shows examples for the positive, intermediate, and negative results for the test. Samples with intermediate and positive results were further subjected to microscopic agglutination test (MAT) as the gold standard test for leptospirosis.

### 2.3. Microscopic Agglutination Test

The microscopic agglutination test (MAT) was performed following the standard method [14]. Serum samples were serially diluted in microtiter plates and live *Leptospira serovars* were added to each well. A panel of 20 reference *Leptospira serovars* included Australis, Autumnalis, Bataviae, Canicola, Icterohaemorrhagiae, Celledoni, Grippotyphosa, Javanica, Pomona, Tarassovi, Semaranga, Djasiman, Lai, Copenhageni, Melaka (IMR LEP 1), Sarawak (IMR LEP 175), Hardjobovis (IMR LEP 22), IMR LEP 127, IMR LEP 113, and IMR LEP 131 as recommended by the Institute for Medical Research (IMR), Malaysia. The plates were then incubated for 2 h at 30 °C and examined using a dark field microscope. Agglutination of at least 50% of the leptospires compared to the control culture was considered as positive. A single MAT titer of 1:400 or more was used as cut off point for seropositive results. This titer was specifically recommended by the IMR as the national reference laboratory, and it was based on the background exposure and seroprevalence of leptospirosis in the country.

### 2.4. Direct Molecular Detection by Real-Time PCR

#### 2.4.1. Extraction of Genomic DNA

The clinical samples were extracted using the Qiagen QIAamp DNA Blood Mini Kit according to the manufacturer’s instructions.

#### 2.4.2. Detection of Leptospira by Real-Time PCR

The amplification of the DNA was conducted in PCR buffer containing 1× SsoAdvanced™ Universal Probes Supermix (Bio-Rad, Hercules, CA, USA), 200 nM forward primer, 200 nM reverse primer, 100 nM probe for both target and internal control genes, 8 µL DNA template with the total volume of 20 µL. The primer and probe sequences were obtained from a previous study [15]. The mixture was subjected into thermal cycler Biorad CFX96 for amplification process; initial denaturation step at 95 °C for 5 min, 50 cycles of 95 °C for 30 s, and 61.3 °C for 30 s. Positivity was indicated by any quantification cycle, Cq value ≤ 40 with the baseline threshold was set at 25 [15]. 

### 2.5. Isolation of Leptospira 

Blood samples in a heparinized tube were collected for isolation of *Leptospira* [12]. Two types of blood samples which were whole blood and deposit from spun plasma were used. For preparation of the deposit from spun plasma, 3 mL of whole blood was transferred into a new tube as described by a previous method [2]. The sample was then centrifuged at 6000 rpm for 3 min to get the deposit. The supernatant was removed and 200 µL of deposit was left for inoculation into the culture media. Whole blood and deposit from spun plasma were cultured in 5 mL modified Ellinghausen McCullough Johnson Harris (EMJH) medium following the standard method [14]. The medium was incorporated with different concentrations of 5’-fluorouracil (5-FU) (0.2 and 0.4 mg/mL of antibiotic concentration) and without antibiotic. 5-FU was added into media because *Leptospira* is resistant to 5-fluorouracil and it suppressed the growth of contaminating bacteria [14]. 

Briefly, 100 µL of whole blood and 200 µL deposit of spun plasma were inoculated into liquid EMJH medium. Cultures were then incubated at 30 °C in incubator shaker (40 rpm) for 6 months and were examined weekly or fortnightly under the dark-field microscope. Reference strains from Research Laboratory, Department of Medical Microbiology and Parasitology, Universiti Sains Malaysia were included as positive controls. If the leptospires were not detectable after 6 months of incubation, the sample was considered to be negative. Meanwhile, presence of at least four *Leptospira* under darkfield microscope was considered as a positive result [16]. 

### 2.6. Molecular Characterization of the Isolates

#### 2.6.1. DNA Extraction

Genomic DNA were extracted from positive EMJH culture using Qiagen DNeasy^®^ Blood and Tissue Kit (Qiagen, Hilden, Germany) according to the manufacturer’s protocol without modifications. The quantity of DNA was measured by Biophotometer (Eppendorf, Germany). The genomic DNA was stored at -20 °C for further uses.

#### 2.6.2. PCR and Visualization of Products

PCR were performed by preparing 25 µL reaction containing 1 mM forward primer (BakII-F: 5’-AGTTTGATCMTGGCTCAG-3’), 1 mM reverse primer (BakII-R: 5’-GGACTACHAGGGTATCTAAT-3’), 12.5 µL of DreamTaq Green PCR Master Mix (Thermo Scientific, Malaysia), 2 µL of DNA template, and 8 µL DNase-free water. Reaction mixtures were then subjected to cycling conditions that consisted of an initial denaturation step at 95 °C for 5 min, followed by 30 amplification cycles of denaturation at 95 °C for 3 s, annealing at 52 °C for 30 s, and extension at 72 °C for 30 s. A final extension step was performed at 72 °C for 5 min. The PCR products were subjected to electrophoresis on a 1.5% agarose gel in 0.5 × TBE buffer at 150 V for 5 min followed by 80 V for 60 min. The gel was imaging under UV light using gel image analyzer (Syngene, Cambridge, UK).

#### 2.6.3. Sequencing and Sequence Analysis

The PCR products were sequenced by First BASE, Selangor, Malaysia. Biological sequence alignment editor (VectorNTI) and the contig result were compared with the GenBank database using BLAST. 

#### 2.6.4. Phylogenetic Analysis of 16S rRNA Gene Sequences

The partial sequences of 16S rRNA gene (*rrs*) were used to analyze the genetic relatedness of the isolates. A phylogenetic tree was created based on aligned sequences using neighbor joining method in MEGA 7 software [17]. The strains used to construct phylogenetic tree were selected based on 16S rRNA gene. The details of strains used were stated at Table 1.

### 2.7. Inhibition of 5’-Fluorouracil Study 

A reference strain of *Leptospira interrogans* serovar Canicola was used in this inhibitory study. The initial concentration of *Leptospira* was 10^8^ cells/mL followed by a serial dilution until 10 cells/mL. *Leptospira* concentration was checked by spectrophotometer at optical density of 0.320 at 420 nm. Approximately at 0.320, *Leptospira* concentration was ≈10^8^ per mL [18] and was confirmed under dark-field microscopy observation detected to be approximately 10^7^ to 10^8^ cell/mL with 100% active motility. 

The EMJH media was added with 5-FU at concentration 0.2 mg/mL. The media containing 5-FU was compared with the media without 5-FU to observe the effect of *Leptospira* growth. 5-FU was used as antibiotic inside the media because *leptospires* is resistant to 5-FU and to suppress the growth of contaminating bacteria [14]. Besides that, EMJH containing 5-FU is broadly used for the selective isolation of *Leptospira*, although it does not inhibit most of the Gram negative bacteria and fungi [19]. The cultures were incubated in incubator shaker at 40 rpm, 30 °C for 7 days and were checked every day until day 7 under dark-field microscope for the viability of *leptospires*. Leptospires concentration was also checked using a tunable microplate reader at optical density of 400 nm. The test was done in duplicate.

## 3. Results 

A total of 109 blood samples were collected from patients with acute febrile illness with not more than 14 days of fever. Two *Leptospira* spp. (*n* = 2/109, 1.85%) were successfully isolated from whole blood and deposit from spun plasma samples. B004 and B208 samples were positive at day 11 and day 7, respectively, in EMJH media without addition of 5-FU. However, the media containing 5-FU did not show any growth of *Leptospira*. 

**Figure 2:**
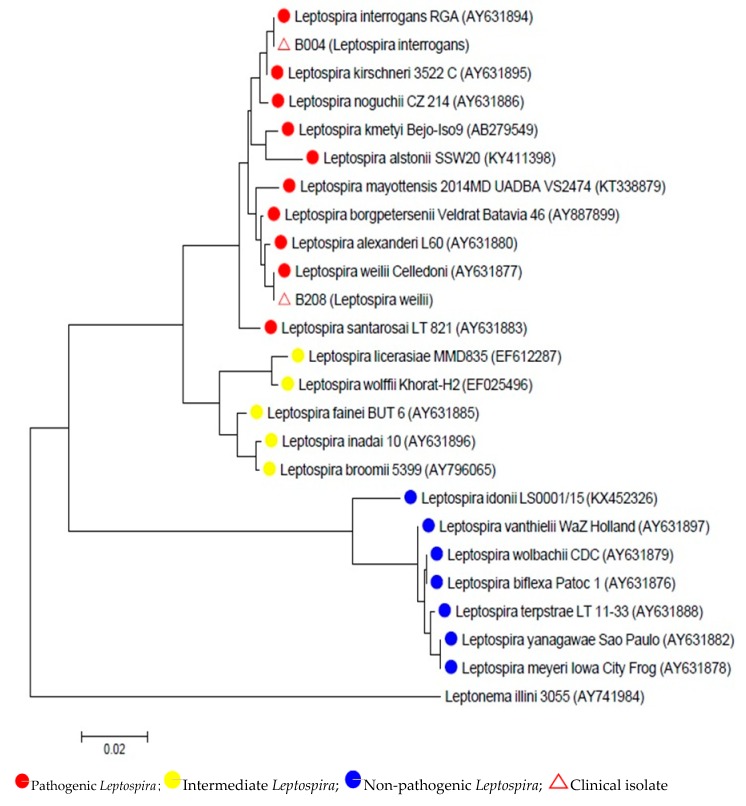
This phylogenetic tree showing evolutionary relationships of taxa of *Leptospira* spp. and the analysis confirmed that B004 and B208 were within pathogenic group.

Following positive growth, *Leptospira* isolates (B004 and B208) were identified by partial sequencing of 16S rRNA gene (*rrs*). The partial sequences of the 16S rRNA gene (*rrs*) showed that B004 and B208 isolates belonged to the pathogenic group of *Leptospira* which were *Leptospira interrogans* and *Leptospira weilli*, respectively. The evolutionary history was inferred using the neighbor-joining method [20]. The optimal tree with the sum of branch length = 0.42237988 is shown in Figure 2. The tree was drawn to scale, with branch lengths in the same units as those of the evolutionary distances used to infer the phylogenetic tree. The evolutionary distances were computed using the maximum composite likelihood method [21] and were in the units of the number of base substitutions per site. All positions containing gaps and missing data were eliminated. There were a total of 480 positions in the final dataset. 

Based on the serological results in Table 2, MAT results were negative for all samples tested (those with intermediate and positive results of *Leptospira* IgM Duo Rapid). Since all titrations were below 1:400, the positive results were not significant. Real-time PCR from direct clinical samples (Table 2) showed that 14.7% (*n* = 16/109) of the samples were positive including B004 and B208 samples. Therefore, leptospirosis was confirmed in 14.7% of the patients with acute febrile illness in this study.

**Figure 3:**
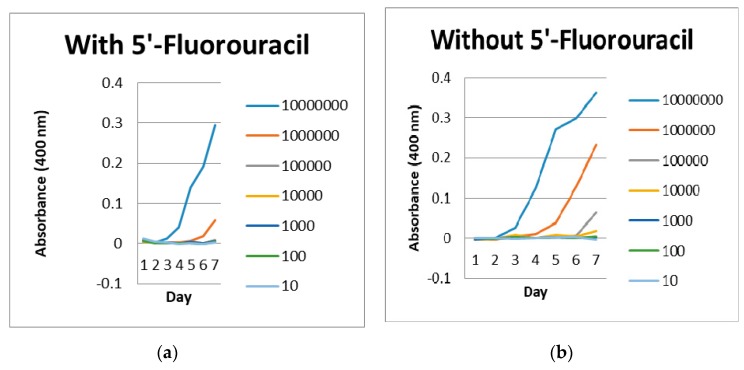
Graph of Leptospira growth with (a) addition of 5’-fluorouracil and (b) without addition of 5’-fluorouracil measured by microplate reader.

In the 5-FU inhibitory study (Table 3), the growth of *Leptospira interrogans* serovar Canicola was detected as early as day 6 in EMJH media without 5-FU at concentration of 10^1^–10^3^ cells/mL and the growth of those cultured in EMJH media with 5-FU was detected at concentration of 10^3^ cells/mL at day 7. At concentration of 10^4^ cells/mL, the isolate was detected as early as day 4 in EMJH media without 5-FU compared to those with 5-FU, the growth was detected at day 7. Using a microplate reader at optical density of 400 nm (Figure 3), *Leptospira* in EMJH media without 5-FU was detected earlier than those in EMJH media with 5-FU.

## 4. Discussion

The diagnosis of leptospirosis is confirmed either by MAT or isolation by culture or nucleic acid detection by PCR from clinical samples. Our study showed that 14.7% (*n* = 16/109) of the patients were diagnosed as leptospirosis based on positive real-time PCR and culture results. All samples were negative by MAT as the titers were below 1:400. Since the samples were collected during the acute phase of the illness, the specific antibody production against *Leptospira* was low and expected to rise during the convalescent stage. Moreover, the sensitivity of the MAT during the acute phase of illness is low and needs a paired serum to confirm the diagnosis [22], however, it was impossible to trace the patients once they returned to their houses. 

Generally, the low isolation of *Leptospira* from blood samples has been the main problem since a long time ago. In this study, 1.85% (*n* = 2/109) of *Leptospira* spp. was successfully isolated from patients presenting with acute febrile illness. Previous studies also reported a low recovery rate for *Leptospira*, but higher than our study, ranging from 7.2% to 9.0% [2,12,13] of the samples. 

Many factors influence the positivity rate of leptospiral culture from blood samples. Blood culture should be taken as soon as possible after the patients’ presentation because *Leptospira* can be isolated in the bloodstream during the leptospiremia phase that occurs during the acute stage of the illness, however the numbers will decrease up to 15 days [23]. Moreover, a previous study reported the maximum duration of fever in patients with positive culture for *Leptospira* in their blood samples was 5 days [2]. Other study also reported that *Leptospira* can be isolated from blood or CSF samples during the first week of illness which is 7–10 days [24]. Thus, the patients with a fever for no more than 14 days were recruited in this study to increase the rate of *Leptospira* isolation. Furthermore, patients with prior antibiotic treatment were excluded from the study since antibiotics quickly remove *Leptospira* from the blood [25]. 

In an ideal circumstance, sample inoculation at the patients’ bedside is highly recommended in order to increase positive growth of *Leptospira* [26]. However, the technique was not applicable in this study due to the logistical problems. To minimize the potential of lower isolation rate, the blood samples were transported to the laboratory immediately at ambient temperature. Nevertheless, it is believed that such procedure was not the reason for the low recovery of *Leptospira* since the longest reported period to recover *Leptospira* from heparinized blood kept at room temperature was 109 days [2]. 

The type of blood specimens and its volume affects the recovery rate for *Leptospira* spp. The standard method is to inoculate one to five drops (100–200 µL) of whole blood directly into EMJH media. The low volume of whole blood used for culturing aims to avoid the inhibition of leptospiral growth by haemoglobin, antibiotics, antibodies, and other blood components factors [27,28]. For the greatest recovery rate, multiple samples should be taken for culture, but this is almost impossible. The type and modification of media used have also been studied. The use of a noble agar base supplemented with 10% rabbit serum (named LVW agar) enables rapid growth, isolation of single colonies, and simple antimicrobial susceptibility testing for *Leptospira* spp. [29]. In addition, *Leptospira* is a fastidious organism and slow grower, making the culture technique very tedious, difficult, and can take up to three months.

Patient factors for low recovery of *Leptospira* might be due to delayed patient presentation and the exact duration of the illness was not disclosed exactly in medical history of patient. To get good samples for isolation study, patients should come for blood investigation as early as possible. Some of them might already take antibiotics prescribed by a general practitioner, but this information was not disclosed during data collection. Some patients with severe illness were unable to disclose the medical history, hence, sole interviews with the spouses, relatives, or companions were carried out. Moreover, this study was conducted at a tertiary teaching hospital located within the capital city of an urban area. Most of the recruited patients in this study stayed within the capital city, hence, they probably went to the nearest private clinic to seek initial treatment for acute febrile illness before attending our hospital at a later phase of the infection.

Isolation of *Leptospira* from clinical samples gives a definitive diagnosis of leptospirosis and also aids in identifying the prevalent species. This study used 16S rRNA gene sequence analysis for species identification of *Leptospira*. 16S rRNA gene sequencing is a common technique for the identification of unknown bacterial isolates, especially those of fastidious organisms such as *Leptospira* species [30]. Both of the isolates namely *L. interrogans* and *L. weilii* belonged to the pathogenic group of *Leptospira.* The phylogenetic tree confirmed the genetic relationships between *Leptospira* with distinct clades comprised of pathogenic, intermediate, and non-pathogenetic species (Table 3). The clinical isolates were comprised in the pathogenic group, however, the species was different and not related. The phylogenetic tree was constructed based on the 16S rRNA gene using one locus therefore the gene cannot be used for differentiation within the species. *L. interrogans* was the most predominant pathogenic *Leptospira* isolated from clinical samples such as blood and urine in human and animals [31,32,33]. In addition, other important serogroups from this species include Australis, Autumnalis, Canicola, Icterohaemorrhagiae, Pomona, Pyrogenes, and Sejroe [34]. Interestingly, *L. weilii* is not commonly isolated from the clinical samples. *L. weilii* strain Celledoni was first isolated from the blood of a patient from North Queensland, Australia [35]. Another previous study reported *L. weilii* serovar Topaz, a newly described serovar isolated from human and animal samples mainly from Far North Queensland [36]. 

In this study, both B004 and B028 isolates that were cultured in EMJH media without addition of 5-FU gave better performance of leptospiral growth than media containing the antibiotic. A previous study found that 5-FU is fatal to various microorganisms but not to *Leptospira* [37]. This compound was used widely to minimize the contamination and to obtain pure primary cultures isolates [37]. However, certain strains of *Leptospira* may be inhibited by 5-FU [38]. Report of a WHO expert group suggested to use both media with and without 5-FU simultaneously for the primary isolation of *Leptospira* spp. [39]. 

In 5-FU inhibitory study, the growth of *Leptospira interrogans* serovar Canicola was detected as early as day 6 in EMJH media without 5-FU at concentration of 10^1^–10^3^ cells/mL and for those with 5-FU, the growth was detected at concentration of 10^3^ cells/mL at day 7. At concentration of 10^4^ cells/mL, the isolate was detected as early as day 4 in EMJH media without 5-FU whereas for those with 5-FU, the growth was detected 3 days later at day 7. In contrast to a previous study, they reported the use of 5-FU at a concentration of 50–1000 µg/mL did not inhibit the growth of Australis, Canicola, and Pomona serovars when those isolates were incubated at 30°C for 7 days [37]. It was also demonstrated that 5-FU was not incorporated into Pomona serovar nucleic acids [37]. Our experimental study suggests that additional of 5-FU at final concentration of 200 µg/mL to EMJH media revealed an inhibitory effect on the growth of the tested strain. Therefore, this experimental study supports our earlier finding that leptospiral culture on EMJH media without addition of 5-FU resulted in a better outcome.

## 5. Conclusions

Leptospirosis was diagnosed in 14.7% of patients with acute febrile illness. Two pathogenic *Leptospira* isolates were successfully cultivated and both of them were genetically not related. Low isolation of *Leptospira* was contributed by many factors. Leptospiral culture on EMJH media without addition of 5-FU resulted in a better outcome. More comprehensive research to optimize the culture method is necessary for improved isolation of *Leptospira* spp. for further epidemiology and molecular characterization studies. 

## Figures and Tables

**Table 1:** Strains studied and their 16S rRNA gene Genbank accession numbers.

Reference Strain	Accession Number
*Leptospira weilii* Celledoni	AY631877
*Leptospira borgpeterseni* Veldrat Batavia 46	AY887899
*Leptospira alexanderi* L60	AY631880
*Leptospira santarosai* LT 821	AY631883
*Leptospira noguchii* CZ 214	AY631886
*Leptospira kirschneri* 3522 C	AY631895
*Leptospira interrogans* RGA	AY631894
*Leptospira vanthielii* Waz Holland	AY631897
*Leptospira biflexa* Patoc 1	AY631876
*Leptospira wolbachii* CDC	AY631879
*Leptospira terpstrae* LT 11-33	AY631888
*Leptospira meyeri* Iowa City Frog	AY631878
*Leptospira yanagawae* Sao Paulo	AY631882
*Leptonema illini* 3055	AY714984
*Leptospira fainei* BUT 6	AY631885
*Leptospira broomii* 5399	AY796065
*Leptospira inadai* 10	AY631896
*Leptospira alstonii* SSW20 *Leptospira idonii* LS 0001/15	KY411398 KX452326
*Leptospira kmetyi* Bejo-Iso9	AB279549
*Leptospira moyottensis* 2014MD UADBA VS2474	KT338879
*Leptospira licerasiae* MMD835	EF612287
*Leptospira wolffii* Khorat-H2	EF025496

**Table 2:** Results of serological and real-time PCR.

*Leptospira* IgM Duo Rapid (*n* = 109)	Microscopic Agglutination Test, Titer (*n* = 31)	Real-Time PCR (*n* = 109)
Intermediate (28) Positive (3) Negative (78)	1:100 (3) 1:50 (6) Negative (22)	Positive (16) Negative (93)

**Table 3:** Microscopic observation under dark-field microscope for culture of *Leptospira interrogans* serovar Canicola with and without 5’-fluorouracil (200 µg/mL).

*Leptospira* Concentration (cells/mL)		Day 1		Day 2		Day 3		Day 4		Day 5		Day 6		Day 7	
		5-FU	No 5-FU	5-FU	No 5-FU	5-FU	No 5-FU	5-FU	No 5-FU	5-FU	No 5-FU	5-FU	No 5-FU	5-FU	No 5-FU
**10^8^**	**I**	**+**	**+**	**+**	**+**	**+**	**+**	**+**	**+**	**+**	**+**	**+**	**+**	**+**	**+**
	**II**	**+**	**+**	**+**	**+**	**+**	**+**	**+**	**+**	**+**	**+**	**+**	**+**	**+**	**+**
**10^7^**	**I**	**+**	**+**	**+**	**+**	**+**	**+**	**+**	**+**	**+**	**+**	**+**	**+**	**+**	**+**
	**II**	**+**	**+**	**+**	**+**	**+**	**+**	**+**	**+**	**+**	**+**	**+**	**+**	**+**	**+**
**10^6^**	**I**	**+**	**+**	**+**	**+**	**+**	**+**	**+**	**+**	**+**	**+**	**+**	**+**	**+**	**+**
	**II**	**+**	**+**	**+**	**+**	**+**	**+**	**+**	**+**	**+**	**+**	**+**	**+**	**+**	**+**
**10^5^**	**I**	**-**	**-**	**-**	**-**	**-**	**-**	**+**	**+**	**+**	**+**	**+**	**+**	**+**	**+**
	**II**	**-**	**-**	**-**	**-**	**-**	**-**	**+**	**+**	**+**	**+**	**+**	**+**	**+**	**+**
**10^4^**	**I**	**-**	**-**	**-**	**-**	**-**	**-**	**-**	**+**	**-**	**+**	**-**	**+**	**+**	**+**
	**II**	**-**	**-**	**-**	**-**	**-**	**-**	**-**	**+**	**-**	**+**	**-**	**+**	**+**	**+**
**10^3^**	**I**	**-**	**-**	**-**	**-**	**-**	**-**	**-**	**-**	**-**	**-**	**-**	**+**	**+**	**+**
	**II**	**-**	**-**	**-**	**-**	**-**	**-**	**-**	**-**	**-**	**-**	**-**	**+**	**+**	**+**
**10^2^**	**I**	**-**	**-**	**-**	**-**	**-**	**-**	**-**	**-**	**-**	**-**	**-**	**+**	**-**	**+**
	**II**	**-**	**-**	**-**	**-**	**-**	**-**	**-**	**-**	**-**	**-**	**-**	**+**	**-**	**+**
**10^1^**	**I**	**-**	**-**	**-**	**-**	**-**	**-**	**-**	**-**	**-**	**-**	**-**	**+**	**-**	**+**
	**II**	**-**	**-**	**-**	**-**	**-**	**-**	**-**	**-**	**-**	**-**	**-**	**+**	**-**	**+**

**+**: positive growth; **-**: negative growth; 5-FU: 5’-fluorouracil.
